# Odontoameloblastoma

**DOI:** 10.4103/0973-029X.80028

**Published:** 2011

**Authors:** Alka Dive, Shubhangi Khandekar, Ashish Bodhade, Akshay Dhobley

**Affiliations:** *Department of Oral & Maxillofacial Pathology, VSPM’S Dental College and Research Centre, Nagpur, India*

**Keywords:** Odontoameloblastoma, mixed odontogenic tumor, ameloblasic dentinoma

## Abstract

Odontoameloblastoma (OA) is an extremely rare mixed odontogenic tumor with both epithelial and mesenchymal components. OA develops from proliferating odontogenic epithelium and mesenchymal tissue. Till date, around 20 cases have fulfilled the histologic criteria of the current World Health Organization (WHO) histologic classification of odontogenic tumors. It affects predominantly young patients with a median age of 20.12 years, and has a predilection for males and occurs in posterior segments of either jaw with slight inclination for mandible. Review of literature shows only three reported cases in the anterior mandible. Here, we report a case of OA in the anterior mandible.

## INTRODUCTION

Odontoameloblastoma (OA) is an extremely rare neoplasm, which is defined by World Health Organization (WHO) and Philipsen & Reichart as follows: “A neoplasm that includes odontogenic ectomesenchyme in addition to odontogenic epithelium that resembles an ameloblastoma (SMA) in both structure and behavior. Because of the presence of odontogenic ectomesenchyme, inductive changes take place leading to the formation of dentin and enamel in parts of the tumor”.[1]

Several names exist for this kind of tumor in the literature, which include odontoblastoma (Thoma, 1970), adamant-odontoma (Shafer *et al*., 1983), calcified mixed odontogenic tumor (Hoffman, 1985), soft and calcified odontoma (Worleyand Mckee, 1972), and ameloblastic odontoma (Hooker, 1967).[2]

The pathogenesis of OA is unknown. One possible explanation is that the mineralized dental tissues are formed as a hamartamatous proliferation in response to inductive stimuli produced by the proliferating epithelium over the mesenchymal tissue.[3]

The term odontoameloblastoma was included in the 1971 WHO classification. It is also known as ameloblastic odontoma, but the term odontoameloblastoma seems to be more appropriate due to the behavior of the tumor like an ameloblastoma rather than as an odontoma. It is an ameloblastoma in which focal differentiation into an odontoma appears. From 1944, when Thoma *et al*. described the first case, till now very few well-documented cases have been reported in the medical literature.[4]

WHO in 1971 deleted the term “ameloblastic odnotoma” from its “histologic typing of odontogenic tumors, jaw cysts and allied lesions”, and subdivided this category into ameloblastic fibroodontoma (AFO) and OA.[5]

OA affects predominantly young patients with a mean age of 20.12 years in the reported cases, appearing up to 59% in patients under 15 years of age. There is a slight male predilection. This tumor usually occurs in the posterior segment of either jaw, with a slight inclination for mandible.[3,4] Only three cases have been reported involving anterior segment of the mandible[4] [Table 1]

**Table 1:** Literature review of odontoameloblastomas (modified from Ref. 2)

S. no.	Author	Age (years)	Sex	Location	Follow-up	Recurrence
1.	Thoma *et al.*	35	Female	Molar - mandible	-	-
2.	Thoma Goldman	20	Male	Ant. max.	2 years	Yes
3.	Silva	31	Female	Ant. max.	-	-
4.	Frissell and Shafer	11	Male	Post. mand.	4 years	Yes
5.	Choukas and Toto	8	Male	Ant. mand.	-	-
6.	Jacobson and Quinn	12	Female	Post. mand.	2 years	No
		20	Female	Post. maxilla	10 years 8 years	No No
7.	Labriola *et al.*	25	-	-		
8.	Gupta and Gupta	51	Male	Post. mand.	18 months	Yes
9.	Takeda *et al.*	11	Female	Ant. maxilla	-	-
10.	Thompson *et al.*	34	Female	Ant. mand.	-	-
11.	Kaugarsand Zussmann	15	Male	Post. max.	7 months	No
12.	Gunbay and Gunbay	11	Male	Ant. max.	7 years	No
13.	Aquado *et al.*	52	Female	Post. max.	17 months	No
14.	Mosqueda *et al.*	25	Male	Post. max.	6 months	No
		15	Male	Post. mand.	1 year	No
		9	Male	Maxilla	3 years	No
15.	Martin Granizo *et al.*	12	Female	Ant. mand.	2 years	No
16.	Palaskar and Nayar[8]	42	Female	Post. mand.	-	-
17.	Mosca *et al*.[9]	22	Female	Ant. max.	6 years	No
		16	Male	Post. mand.	6 months	No
18.	Sapru *et al*.[10]	36	Male	Post. mand.	1 year	No

Post- posterior, Ant- anterior, Mand- mandibular, Max- maxillary

The tumor exhibits growth characteristics similar to ameloblastoma and presents centrally destructive lesions. Symptoms include a slow progressive swelling of the alveolar plates, dull pain, an altered occlusion, delayed eruption or impacted teeth.[2,4,6]

Radiological examination usually reveals a multilocular radiolucency with radiopaque areas resembling mature dental tissue. It commonly exhibits a well-defined margin, displacing the surrounding erupted teeth rather than producing root resorption.[4]

Treatment wise, OA should be treated by wide excision and closely followed up for at least 5 years,[3] as there is a high rate of recurrence.[2,7]

The exact incidence of this neoplasm is difficult to determine as the current information on this unusual lesion comes from isolated and sporadic case reports.[3] In many cases, OA is often confused with compound or complex odontome.[4]

The aim of this article is to present a rare case of OA in the anterior mandible.

## CASE REPORT

A 47-year-old male patient reported to the Department of Oral and Maxillofacial Pathology, with the chief complaint of a rapidly growing swelling on the back side of the anterior lower jaw since 3 months. The patient gave the history of similar lesion at the same site which recurred twice in the last 7 years, for which he underwent surgical procedures. No record was available with the patient of the previous treatments.

**Figure 1:**
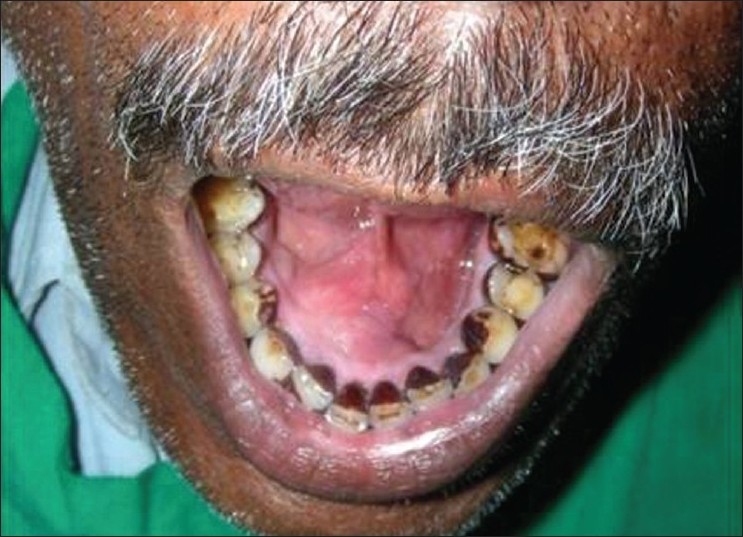
Mucosal colored swelling of approximately 3 cm in diameter on the lingual aspect of mandible on the right side, extending from 32 to 44

**Figure 2:**
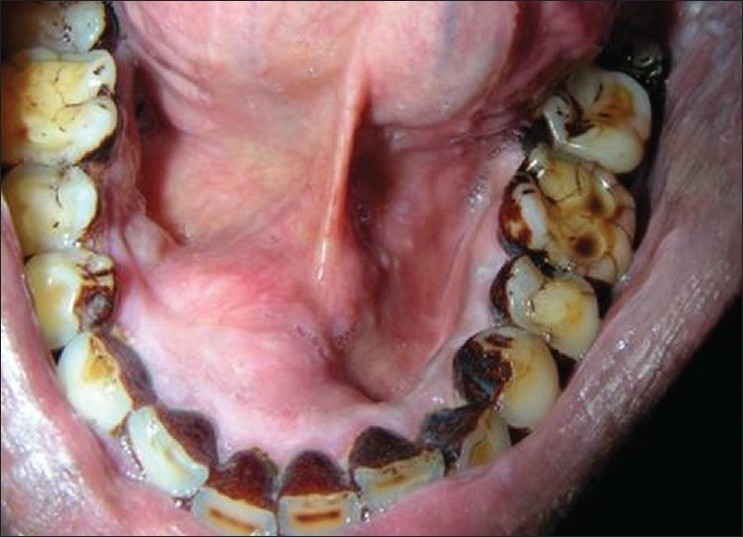
Swelling of approximately 3 cm in diameter on the lingual aspect of mandible on the right side, extending from 32 to 44

**Figure 3:**
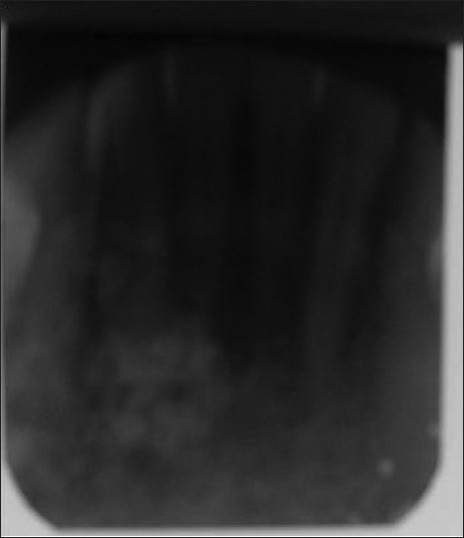
IOPA shows a diffuse radiolucent area with scattered flecks of radiopacities at the apical region of 41–43

**Figure 4:**
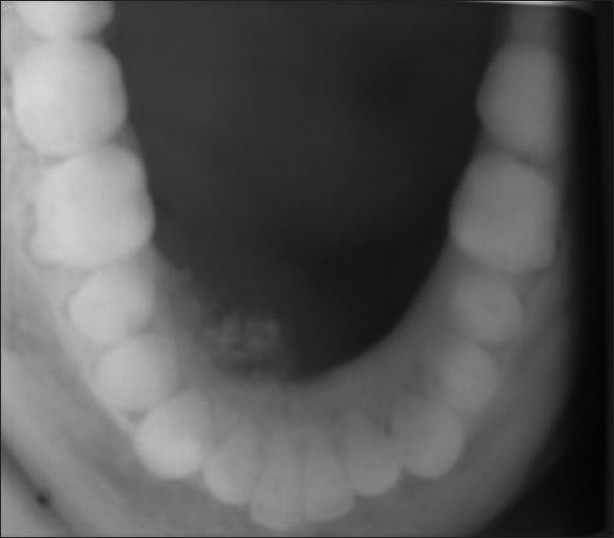
Mandibular occlusal radiograph reveals diffuse foci of radiopacities scattered in a radiolucent area with expansion of lingual cortical plate

Intraoral examination revealed a mucosal colored swelling of approximately 3 cm in diameter on the lingual aspect of mandible on the right side, extending from 32 to 44. The swelling was firm, non-tender, non-fluctuant, non-compressible [Figures 1 and 2]. Intraoral periapical radiograph revealed a diffuse radiolucent area with scattered flecks of radiopacities at the apical region of 41–43 [Figure 3]. Mandibular occlusal radiograph revealed diffuse foci of radiopacities scattered in a radiolucent area with expansion of lingual cortical plate [Figure 4].

A provisional diagnosis of calcifying epithelial odontogenic tumor was considered. Routine hematological investigations were within normal limits. The tumor was carefully excised and the specimen was sent for histopathologic examination.

**Figure 5:**
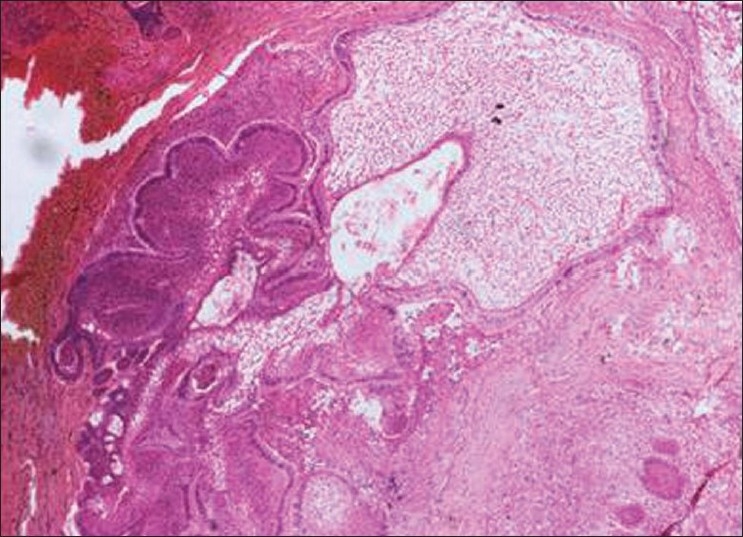
Odontogenic epithelial cells arranged in the form of follicles and stellate reticulum like cells in the center which are surrounded by ectomesenchymal cells (4×)

**Figure 6:**
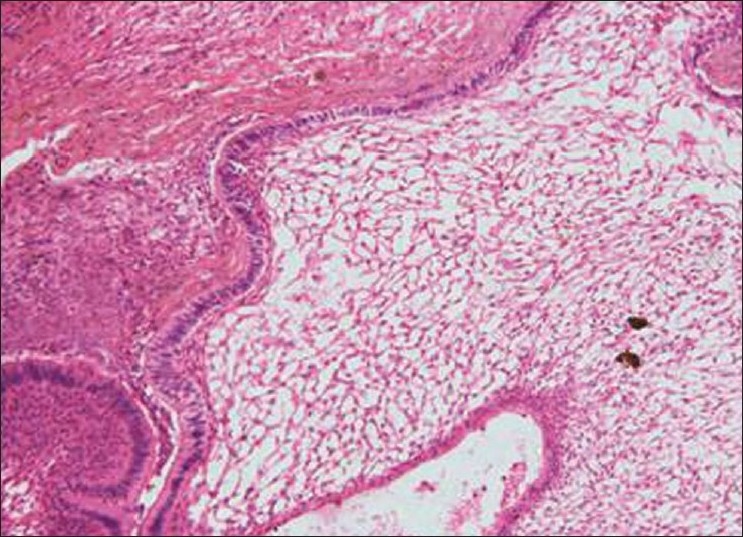
tall columnar ameloblast like cells on the periphery with stellate reticulum like cells at the center and surrounded by condensed mesenchymal cells (10×)

**Figure 7:**
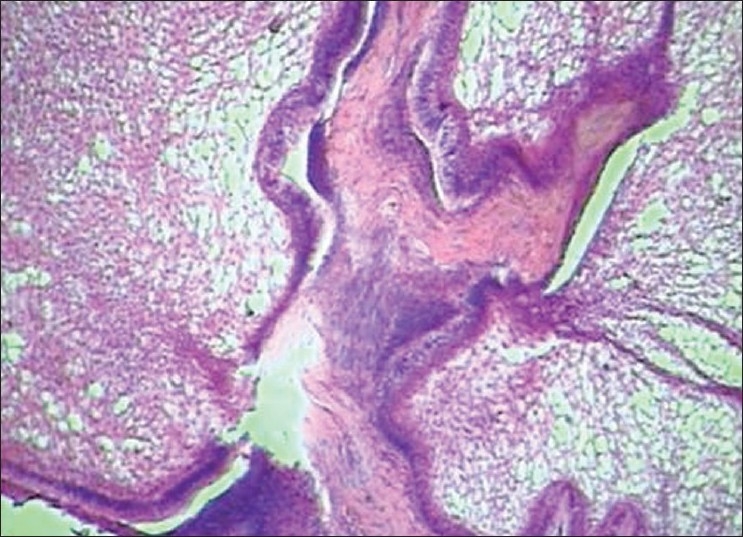
Neoplastic odontogenic epithelium in the form of large follicles (10×)

**Figure 8:**
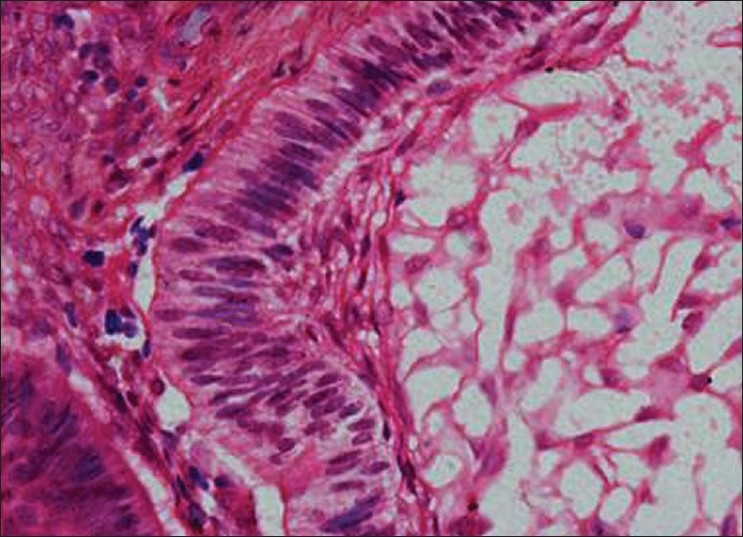
Tall columnar ameloblasts like cells showing nuclear palisading, reversal of polarity and stellate reticulum like cells (40×)

**Figure 9:**
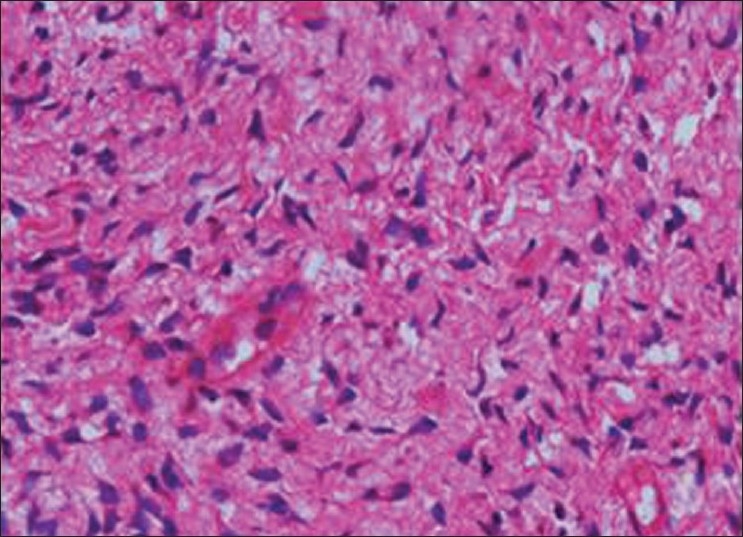
Dense ectomesenchymal cells present in the connective tissue stroma (40×)

**Figure 10:**
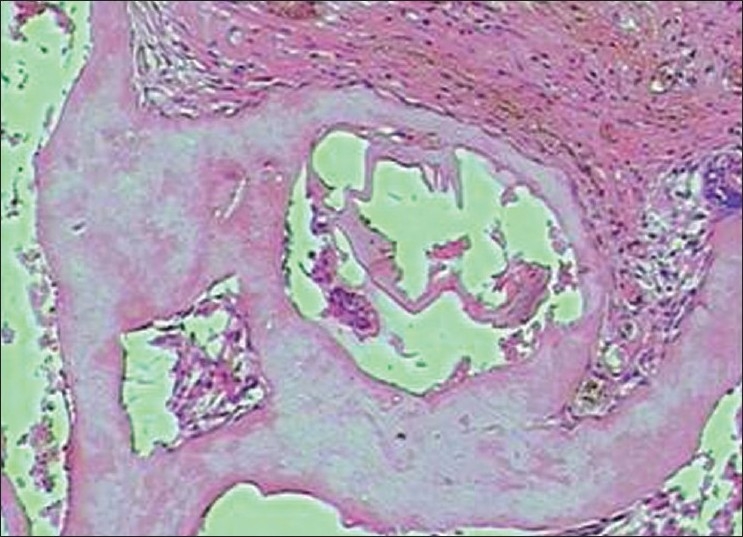
large masses of dysplastic dentin arranged in a haphazard pattern. (10×)

**Figure 11:**
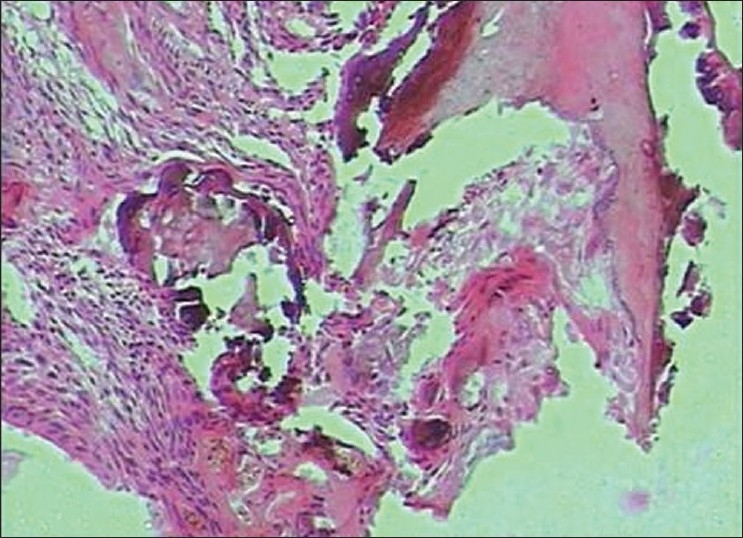
Irregular masses of dysplastic dentin, areas of calcification and stromal connective tissue(10×)

Histopathology showed the following: odontogenic epithelial cells arranged in the form of follicles and stellate reticulum like cells in the center which are surrounded by ectomesenchymal cells (4×) [Figure 5]; tall columnar ameloblast like cells on the periphery with stellate reticulum like cells at the center and surrounded by condensed mesenchymal cells (10×)[Figure 6]; neoplastic odontogenic epithelium in the form of large follicles (10×) [Figure 7]; Tall columnar ameloblasts like cells showing nuclear palisading, reversal of polarity and stellate reticulum like cells (40×) [Figure 8]; dense ectomesenchymal cells present in the connective tissue stroma (40×) [Figure 9]; large masses of dysplastic dentin arranged in a haphazard pattern [Figure 10]; and irregular masses of dysplastic dentin, areas of calcification and stromal connective tissue [Figure 11]. The diagnosis of OA was made. The postoperative recovery was uneventful, and after 2 years, the patient is well with no evidence of recurrence.

## DISCUSSION

OA is an aggressive odontogenic tumor. The lesion has generated much confusion and controversy in the literature as evidenced by the reporting of developing odontomas, AFOs and OA under the terms ameloblastic odontomas, admantoodontona and soft and calcified odontomas. To clarify the confusion, WHO deleted ameloblastic odontoma from its “histologic typing of odontogenic tumors, jaw cysts and allied lesions”, and subdivided this category into AFOs and odontoameloblastoma. This separation was based on the differences in the clinical and histological behavior of the lesions.

Review of literature of 22 cases of OA showed the histologic criteria of unequivocal ameloblastoma, mature connective tissue, fragments of malformed calcified dental structures [Table 1].

OA is a very rare mixed odontogenic neoplasm characterized by the simultaneous occurrence of an ameloblastoma and a compound or complex odontoma in the same tumor mass. The epithelial proliferation forms islands or intermingled cords that produce the follicular or the plexiform patterns typical of ameloblastoma, but unlike conventional ameloblastoma, these induce the production of mineralized dental tissues on the adjacent mesenchymal cells and may respond to these changes with the production of enamel.[3]

This tumor is not exclusive of the human race and has also been described in sheep, monkeys, cats, and rats.[4] Some OAs may contain ghost cells. It is important to exclude other tumors that also contain such cells, namely, Calcifying dontogenic Cysts and odontogenic ghost cell tumors.[3]

Even though very few cases have been reported, it is suggested that OA possesses clinical and microscopic features that allow differentiating it from typical ameloblastomas and odontomas. First, it tends to occur with equal frequency in the mandible and maxilla, while ameloblastoma is prevalent in the mandible. Secondly, OA tends to produce bone expansion similar to ameloblastoma, while odontoma seldom produces swelling of the affected region. Some OAs present intermittent or dull pain which is not usually referred in other benign odontogenic tumors. A case of peripheral OA has been reported by Palaskar in the region of 37, 38.[8]

Histologically, it is difficult to differentiate between OA, AFO and ameloblastic fibrodentinoma (AFD).[5] AFO and AFD are slow-growing lesions with no propensity for bony expansion, mostly associated with an unerupted tooth.[1] AFD and AFO can be treated with enucleation without much danger of recurrence. OA, on the other hand, expands by infiltrating between bony trabeculae, has a high rate of recurrence and should be aggressively treated like conventional ameloblastoma and close follow-up should be done. OA is unusual in that a relatively undifferentiated neoplastic tissue is associated with highly differentiated tissue, both of which show recurrence after adequate removal. Many structures resembling normal or atypical tooth germs may be found with or without the presence of calcified dental tissues.[9]
